# Visitor Characteristics and Museum Fatigue: A Case Study at the ETRU Museum in Rome

**DOI:** 10.3390/brainsci16020225

**Published:** 2026-02-13

**Authors:** Claudio Zavattaro, Emanuele Cirillo, Hilary Serra, Gianluca D’Agostino, Paolo Dabove, Michela Benente, Valeria Minucciani, Anna Berti, Raffaella Ricci

**Affiliations:** 1 Space, Attention, and Action (SAN) Lab, Department of Psychology, University of Turin, Via Verdi 10, 10124 Turin, Italy; claudio.zavattaro@unito.it (C.Z.); emanuele.cirillo@unito.it (E.C.); hilary.serra@unito.it (H.S.); annamaria.berti@unito.it (A.B.); 2 Department of Physics, University of Trento, Via Sommarive 14, 38123 Trento, Italy; 3 Department of Architecture and Design, Politecnico di Torino, Viale Pier Andrea Mattioli 39, 10125 Turin, Italy; gianluca.dagostino@polito.it (G.D.); michela.benente@polito.it (M.B.); valeria.minucciani@polito.it (V.M.); 4 Department of Environment, Land, and Infrastructure Engineering, Politecnico di Torino, Corso Duca degli Abruzzi 24, 10129 Turin, Italy; paolo.dabove@polito.it

**Keywords:** museum fatigue, visitors, physiology, emotional state, tracking

## Abstract

**Highlights:**

**What are the main findings?**

- The emergence of museum fatigue leads to decreased viewing time, but also to increased heart rate.
- While viewing time trends are consistent between individuals, heart rate trends are different depending on individual characteristics.

**What are the implications of the main findings?**

- Heart rate may be employed as an implicit measure of museum fatigue.
- Physiological measures must be considered when investigating individual characteristics linked to museum fatigue.

**Abstract:**

Background/Objectives: Museum fatigue decreases visitors’ interest due to environmental, social, and personal factors. However, it remains unclear whether physiological parameters can capture museum fatigue, and whether personal factors contribute to psychophysiological changes associated with museum fatigue. Methods: To fill these knowledge gaps, 61 participants visited the ETRU museum in Rome while their position and heart rate (HR) values were continuously recorded. Emotional state was rated after the visit. Time-series analyses assessed trends in viewing time and HR across the full sample and in three clusters defined by personal factors, with correlations examining associations among visit time, HR, and emotional states. Results: Overall, viewing time decreased, while HR increased during the visit. Emotional state correlated positively with visit time, but negatively with HR. The viewing time decrease was consistent across clusters, while HR trends and correlations differed. Conclusions: These findings confirmed that environmental characteristics induce museum fatigue in the visitors and showed that heart rate may be employed as an implicit measure of museum fatigue. In addition, this study revealed that personal factors can modulate the emergence of this phenomenon.

## 1. Introduction

Museum fatigue is a well-recognized phenomenon often experienced by visitors as they progress in a museum visit. Conceptually defined as a “collection of phenomena that represents predictable decreases in visitor interest and selectivity” [1,2], museum fatigue can compromise both educational and recreational purposes of museum experiences. Since the term was coined by Gilman [3], many studies investigated museum fatigue in different contexts to understand its components [1] and contributing factors [4]. In addition, countermeasures that mitigate its emergence have been studied, such as the introduction of virtual reality (VR), augmented reality (AR) or multisensory experiences to enhance and/or maintain visitor interest [5,6]. These studies highlighted how personal (background knowledge, motivation, and interest), environmental (visit length, number of displayed artifacts, presence of break rooms), and social (interactions with other visitors) factors can play a role in the emergence of museum fatigue [4,7]. However, inconsistent results have been found. While pioneering studies have demonstrated the presence of museum fatigue [8,9], others have failed to observe fatigue effects even during long museum visits (e.g., [10]). There are two potential explanations for these inconsistent results. The first explanation is related to how this very complex phenomenon is measured. Indeed, previous studies mainly focused on behavioral changes—e.g., reduction in viewing time of areas or artifacts as the visit progresses—and on subjective reports about the visit experience—e.g., emotional state and evaluation of museum exhibition (for a review, see [1]). However, while behavioral changes are measured during the visit, subjective scales are typically administered after the visit. Consequently, visitors’ responses rely on retrospective evaluations based on memories, which do not capture the psychological changes that occurred during the visit [11]. The second explanation relies on the variability of environmental factors of the visit and/or of personal factors of visitors across studies. Indeed, there is evidence that environmental factors—the number of routes, the quantity of exhibited artifacts, the duration of the visit, or the presence of breaks during the visit—play a fundamental role in the emergence of museum fatigue [1,4,11,12]. Conversely, while it is recognized that demographic characteristics and interest in artistic experiences are important in generating museum fatigue [4], to the best of our knowledge, no experimental study has specifically investigated whether behavioral and psychophysiological changes during a museum visit differentially emerge when visitors’ individual characteristics are taken into account.

To fill these gaps, in this study, 61 healthy volunteers visited the National Etruscan Museum (ETRU) in Rome, which provided an ideal context to examine how environmental factors contribute to the emergence of museum fatigue, due to the presence of a great number of artifacts displayed on an accessible area of more than 2000 m^2^. To investigate the presence of museum fatigue during the visit, the participants’ location inside the museum was collected via a tracking system, and the viewing time of museum areas was analyzed. Concomitantly, heart rate values—an established measure of visitors’ arousal [13,14,15,16]—were continuously recorded to examine whether they may represent an implicit measure of museum fatigue, offering a novel implicit way to capture this complex phenomenon. Emotional state was assessed with a subjective scale after the visit ended to investigate potential associations between this factor, visit time, and heart rate. Demographic information and information on the interest of participants in visiting museums were collected before the visit with the aim of exploring whether visitors’ personal characteristics may have modulated both viewing time and psychophysiological state during the visit. We predicted decreased viewing time and increased arousal as the visit progressed. In addition, we predicted that visit time and mean heart rate would be associated with negative emotional state ratings. Lastly, these trends and associations were expected to be modulated by visitors’ personal characteristics.

## 2. Materials and Methods

### 2.1. Participants

Sixty-one healthy participants signed the informed consent documentation and participated in the study, but 5 were excluded from the analysis due to missing data, and another 11 due to the visit duration limit for museum closing hours. Therefore, only data from 45 participants were analyzed (29 F/16 M). Demographic information (age, gender, education) was collected before the visit (see Table 1). In addition, the number of museums visited per year was collected before the visit to classify the participants as “non-public” (0–2 museums per year), “general public” (3–5 per year), or “museumgoers” (more than 5 per year). The study was approved by the Ethical Committee of the University of Turin and followed the Declaration of Helsinki.

### 2.2. Museum Visit

**Figure 1:**
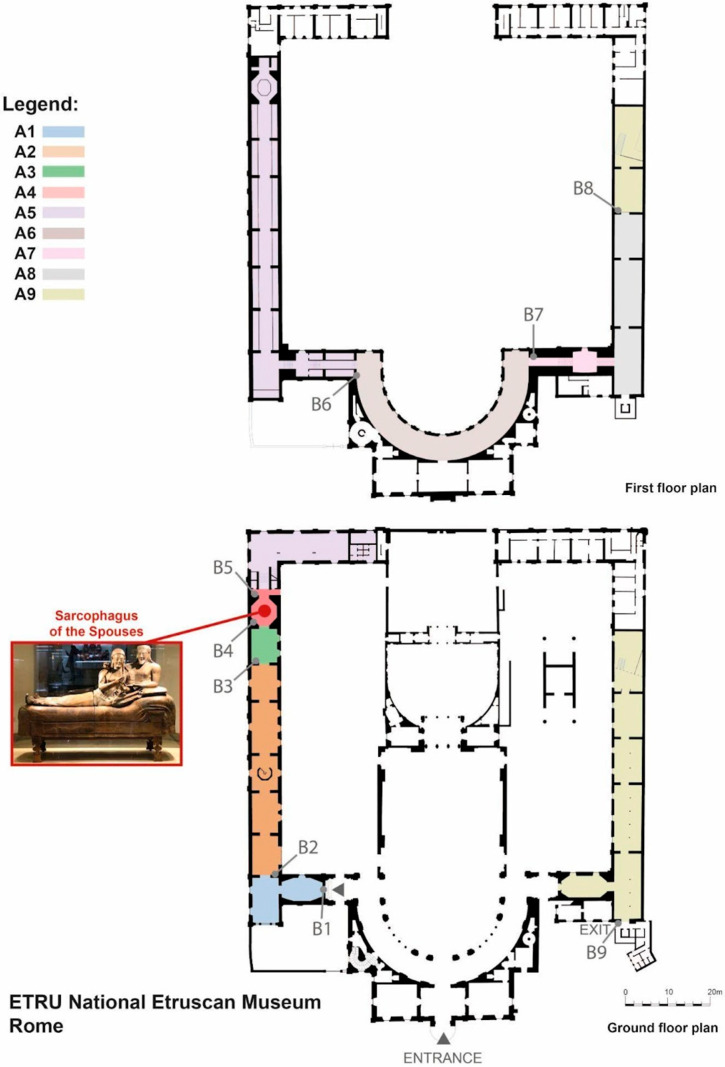
Museum map with areas depicted with different colors (from A1 to A9). Each beacon is represented by its name (from B1 to B9) referring to its position on the museum map.

The visit was held at the National Etruscan Museum (ETRU, Museo Nazionale Etrusco di Villa Giulia) in Rome, which has an accessible area of more than 2000 m^2^ and a large number of artifacts exhibited in 192 showcases of various sizes. The museum building consists of two wings, each with two floors, connected to the first floor by a hemicycle. The museum was conceptually divided into nine areas for analytical purposes. The ground floor of the left wing comprised four areas: the main entrance room (A1), the long corridor after the first right turn (A2), the room leading to the main artifact—the Sarcophagus of the Spouses—(A3), and the Sarcophagus of the Spouses room (A4). The first floor of the left wing was treated as a single area (A5), as was the hemicycle connecting the two wings (A6). The first floor of the right wing was divided into two areas: the room where the Bisentium vase was exhibited (A7) and most of the rest of the floor (A8). The ground floor of the right wing was considered as a single area (A9). The museum map and areas are depicted in Figure 1. Considering the building structure, the visit was linear and with no multiple routes.

### 2.3. Outcome Measures

To track participants’ position in each museum area, a system consisting of Eddystone beacons and a Raspberry Pi 4B (a single-board computer developed by Raspberry Pi Ltd., Cambridge, UK, raspberrypi.org) was used. Eddystone beacon parameters were set in order to transmit Bluetooth Low Energy (BLE) signals in every direction at 10 Hz frequency and in a 10 m range (−16 dBm transmission power). The Raspberry Pi 4B continuously searched BLE signals at 1 Hz frequency by means of a running Python (version 3.10) script and stored the datetime and the beacon name of each detection. The viewing time (in seconds) of each area was calculated by subtracting the area exit time from the area entry time. Then, due to the substantial difference in the dimensions of museum areas (see Table 2), the ratio between viewing time and surface (s/m^2^) of each area was calculated to ensure comparability between areas.

Regarding physiological measures, an Empatica E4 (Empatica Srl, Milan, Italy) wristband was used to continuously record participants’ blood volume pulse (rhythmic variation in blood volume within blood vessels, BVP) at 64 Hz using the photoplethysmography technique. Then, the E4 wristband automatically derived heart rate (HR) at 1 Hz, interpreted as an index of sympathetic nervous system activity linked with participants’ arousal state. Intra-participant standardization was employed on HR values to reduce the interindividual variability of data. To do so, the z-scores of the nine museum areas were calculated based on the individual HR mean and standard deviation.

For the psychological measures, participants completed a questionnaire in which they had to state their level of relaxation, involvement, calmness, and enthusiasm during the visit (with a −2/+2 range as in [17]). This scale was used to better interpret physiological results, as an increase in HR may be interpreted both as a negative stress response or as a positive emotional response to the museum visit.

### 2.4. Experimental Procedure

Before the visit, participants signed the informed consent form and completed the demographic questionnaire. Then, they wore the Empatica E4 wristband on their non-dominant wrist and the position tracking system around their neck. Before starting the visit, they completed a 5 min rest period in which the physiological baseline was collected. Then, they completed the visit without any restriction, during which the entry and exit times in each museum area were detected. Also, their physiological measures were continuously collected. After the visit, they completed the emotional state questionnaire.

### 2.5. Data Analysis

Data analyses were performed using R (version 4.3.3). To cluster participants according to their demographics and interest in museums, a three-step cluster analysis was conducted. At first, we employed multiple correspondence analysis (MCA) to assess the relationships between the categories of four variables (gender, age, education, and public). After MCA, the relationships between categories were represented by chi-square distances on an n-dimensional geometrical plane interpreted as Euclidean distances [18].

The second step is aimed at defining the most appropriate number of clusters. To do so, we employed hierarchical cluster analysis (HCA) with Ward’s method on the Euclidean distances between categories. This method is mainly used with small samples and aims at minimizing the within-clusters variance while maximizing the between-clusters variance [18,19]. After participants were assigned to their cluster by means of HCA, in the third step, we employed the v-test to assess the most represented categories in each cluster. Indeed, the v-test compared the number of participants representing each category in a specific cluster to the number of participants representing that category in the total sample [18]. A v > 2 indicated overrepresentation of that category in the cluster compared to the whole sample, while a v < −2 indicated underrepresentation.

To investigate the presence of behavioral and physiological changes during the visit, the viewing time (in s/m^2^) and zHR values of museum areas were treated as spatial time series, which represent time series collected at fixed spatial locations [20]. The Mann–Kendall test was used to assess the presence of monotonic trends in viewing time and zHR with visit progression. The same analysis was then conducted on each cluster to assess the presence of different trends between clusters in both viewing time and zHR. Lastly, associations between visit time, mean HR, and emotional state ratings were assessed by means of Spearman correlations both in the whole sample and in each cluster.

## 3. Results

### 3.1. Clustering Analysis Results

**Figure 2:**
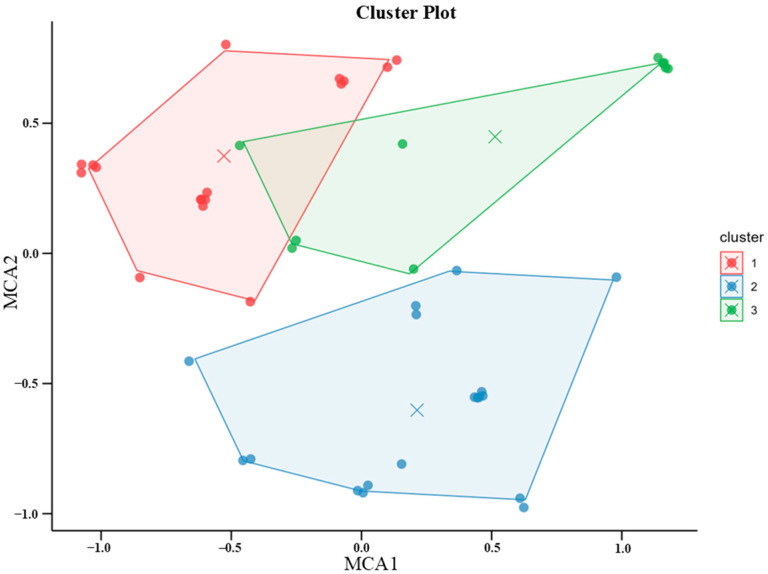
Cluster plot showing the distribution of participants across three clusters based on MCA dimensions 1 (MCA1) and 2 (MCA2). Participants are represented by jittered circles colored according to the assigned cluster. Centroids of each cluster are highlighted with colored crosses.

Results from the MCA revealed that the overall variance of the data could be displayed in six dimensions. However, since the first two dimensions explained the highest amount of variance (27% and 21.9%, respectively), the relationships between categories were plotted in a two-dimensional plane, where each variable category was a point in the plot indicating how much it contributed to the variance of each of the two dimensions. HCA was then applied to the Euclidean distances between variables. Visual inspection of the resulting dendrogram indicated that a three-cluster solution was the most appropriate for the sample. Therefore, each participant was assigned to one of three clusters (see Figure 2): Cluster 1 (*n* = 19), Cluster 2 (*n* = 16), and Cluster 3 (*n* = 10).

Results from the v-test revealed that Cluster 1 was significantly overrepresented by adults (66.67% of the total number of participants representing this variable, v = 2.462, *p* < 0.014) classified as the general public (100%, v = 6.335, *p* < 0.001), Cluster 2 by participants with higher education (57.69%, v = 3.2, *p* = 0.001) classified as museumgoers (89.47%, v = 6.4, *p* < 0.001), and Cluster 3 by participants classified as non-public (100%, v = 6.292, *p* < 0.001). In Table 3, the distribution of each demographic variable within each cluster is depicted.

### 3.2. General Results

**Figure 3:**
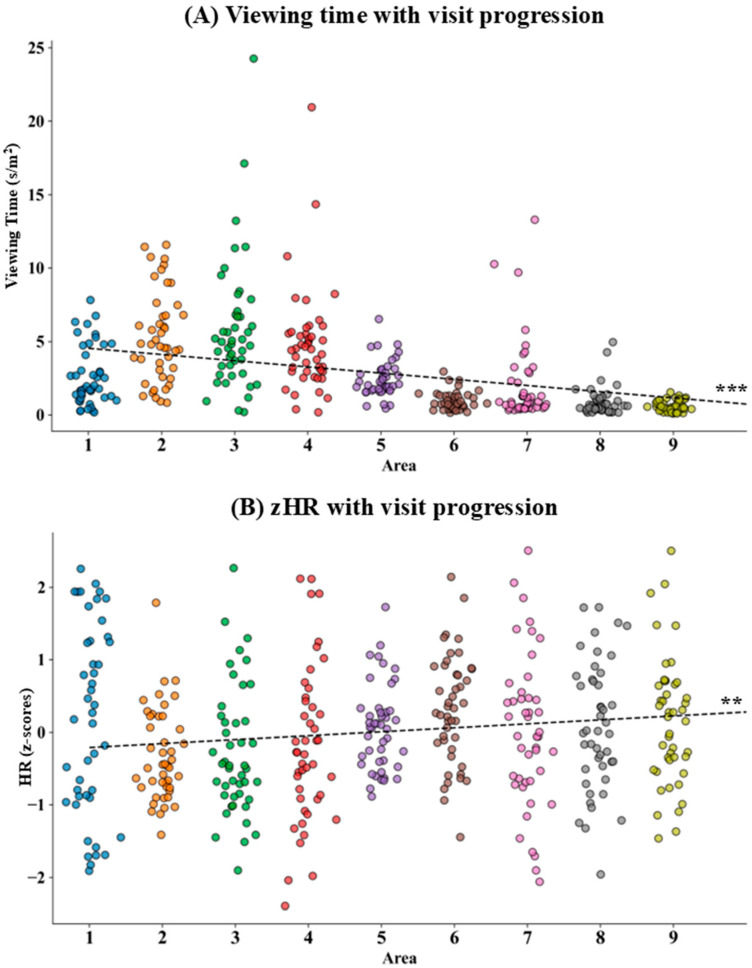
Distribution plot of participants: (**A**) viewing time and (**B**) heart rate (HR, z-values) for each area (A1–A9), with lines depicting linear trends. Participants are represented by jittered circles colored according to the related museum area. ** = *p* < 0.01; *** = *p* < 0.001.

A Mann–Kendall test on viewing time and zHR during the visit revealed a significant negative trend in viewing time (*τ* = −0.437, *p* < 0.001; see Figure 3A) and a significant positive trend in HR (*τ* = 0.092, *p* = 0.005; see Figure 3B) as the visit progressed.

**Figure 4:**
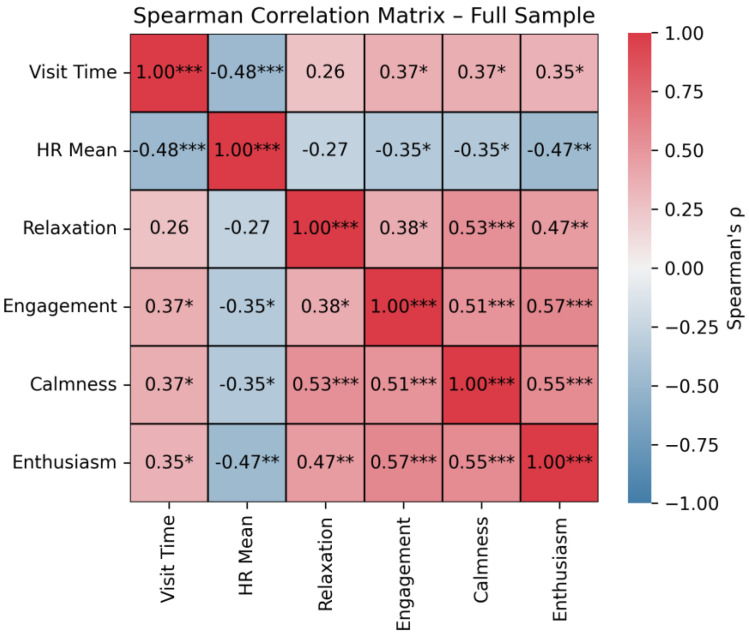
Correlation matrix between visit time, heart rate and emotional state adjectives. * = *p* < 0.05; ** = *p* < 0.01; *** = *p* < 0.001.

Spearman correlations revealed a significant negative correlation between visit time and mean HR (ρ = −0.481, *p* < 0.001). In addition, significant positive correlations between visit time and involvement (ρ = 0.366, *p* = 0.013), calmness (ρ = 0.373, *p* = 0.012), and enthusiasm (ρ = 0.346, *p* = 0.02) ratings were observed. On the other hand, significant negative correlations were found between mean HR and involvement (ρ = −0.346, *p* = 0.02), calmness (ρ = −0.355, *p* = 0.017), and enthusiasm (ρ = −0.474, *p* = 0.001) ratings. A correlation matrix for all variable pairs is depicted in Figure 4.

To examine the robustness of these trends and correlations, a Mann–Kendall test and Spearman correlation were employed at the individual level. The results showed that a decrease in viewing time emerged in 44 participants (97.78%), while an increase in HR appeared in 31 participants (68.89%). Moreover, 34 participants (75.56%) showed a negative correlation between viewing time and HR, but the correlation was significant in 17.78% of the sample. A table with the individual trends and correlations can be found in Appendix A (Table A1).

Lastly, an exploratory analysis was conducted to examine whether the baseline HR was related to the viewing time or zHR trends. Two linear regression models were employed between the Mann–Kendall *τ* values and the baseline HR. For these analyses, no significant results emerged (*p* > 0.05).

### 3.3. Cluster Results

**Figure 5:**
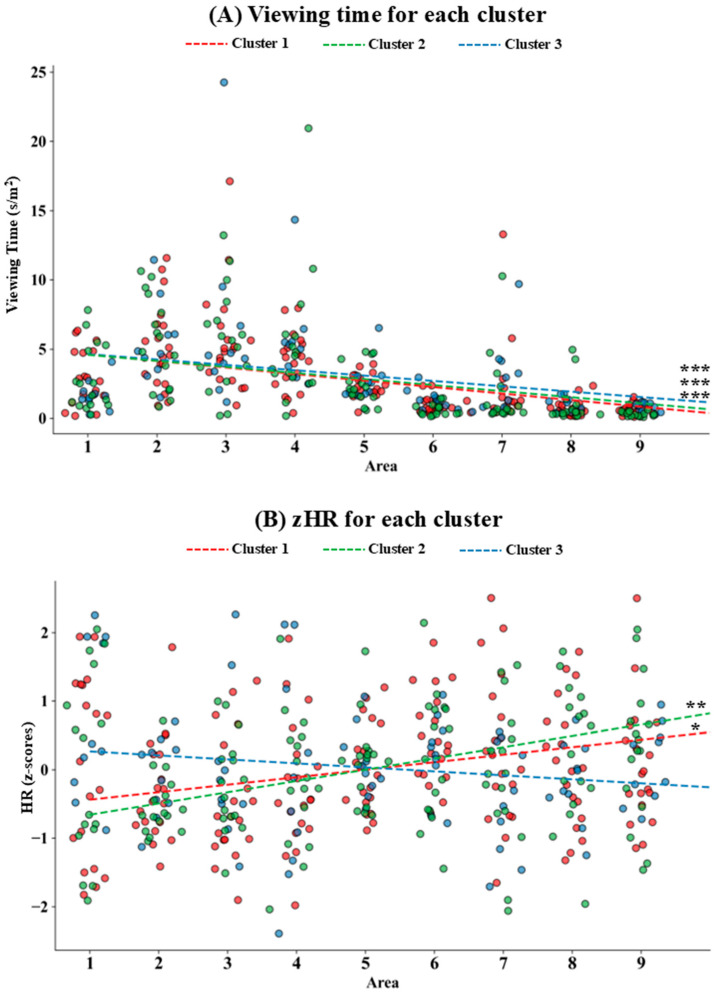
Distribution plots of (**A**) viewing time and (**B**) heart rate (z-values) across each area. Participants are represented by jittered circles colored according to the assigned cluster, while the colored lines represent the trend for each cluster. * = *p* < 0.05; ** = *p* < 0.01; *** = *p* < 0.001.

A Mann–Kendall test on viewing time and zHR was employed for each cluster. Regarding viewing time, a significant negative trend was observed in all clusters (Cluster 1: *τ* = −0.466, *p* < 0.001; Cluster 2: *τ* = −0.45, *p* < 0.001; Cluster 3: *τ* = −0.391, *p* < 0.001; see Figure 5A). Conversely, a significant positive trend in zHR was observed in Cluster 1 (*τ* = 0.116, *p* = 0.024) and Cluster 2 (*τ* = 0.168, *p* = 0.003), but not Cluster 3 (*τ* = −0.043, *p* = 0.549; see Figure 5B).

**Figure 6:**
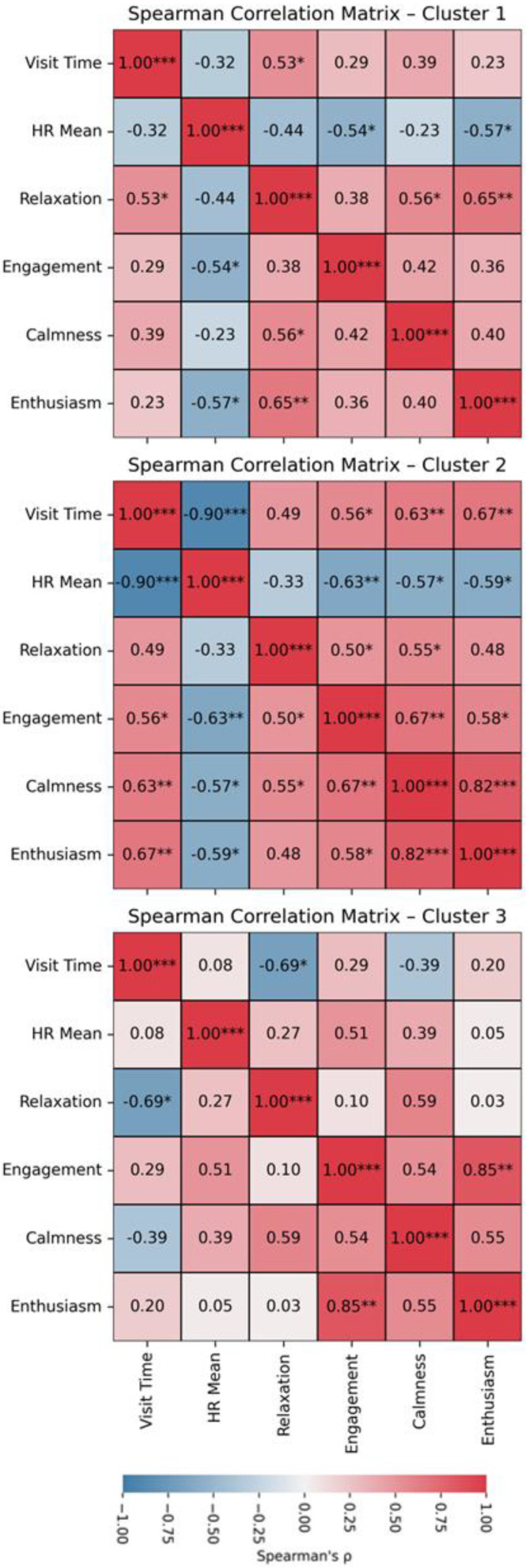
Correlation matrix for each cluster between visit time, mean heart rate and emotional state adjectives. * = *p* < 0.05; ** = *p* < 0.01; *** = *p* < 0.001.

Also, correlation analyses were employed in each cluster. For Cluster 1, a significant positive correlation was observed between visit time and relaxation (ρ = 0.528, *p* = 0.02), and significant negative correlations were found between mean HR and both engagement (ρ = −0.543, *p* = 0.016) and enthusiasm ratings (ρ = −0.569, *p* = 0.011). For Cluster 2, a significant negative correlation was found between visit time and mean HR (ρ = −0.902, *p* < 0.001) and significant positive correlations were observed between visit time and involvement (ρ = 0.563, *p* = 0.023), calmness (ρ = 0.633, *p* = 0.008), and enthusiasm (ρ = 0.671, *p* = 0.004) ratings. In addition, significant negative correlations were found between mean HR and involvement (ρ = −0.629, *p* = 0.009), calmness (ρ = −0.572, *p* = 0.021), and enthusiasm (ρ = −0.59, *p* = 0.016) ratings. For Cluster 3, a significant negative correlation between visit time and relaxation ratings (ρ = −0.688, *p* = 0.028) was found (see Figure 6).

The robustness of these results was assessed for each cluster by employing a Mann–Kendall test and Spearman correlation at the individual level). The results showed that a negative trend in viewing time emerged in 19 participants in Cluster 1 (100%), 16 participants in Cluster 2 (93.75%) and 10 participants in Cluster 3 (100%). Moreover, a positive trend in HR appeared in 14 participants in Cluster 1 (73.68%), 12 participants in Cluster 2 (75%), and 5 participants in Cluster 3 (50%). Regarding the negative correlation between viewing time and HR, 16 participants in Cluster 1 (84.21%), 12 participants in Cluster 2 (75%), and 6 participants in Cluster 3 (60%) showed this correlation, but it was significant in 15.79%, 25%, and 10% of participants, respectively.

**Figure 7:**
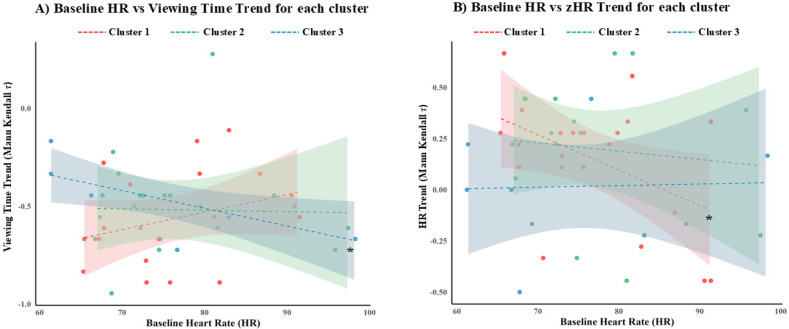
Linear regression plot for each cluster between baseline HR, (**A**) viewing time trend, and (**B**) zHR trend. Participants are represented by jittered circles colored according to the assigned cluster; the colored lines represent the regression slope for each cluster, and the colored areas the confidence interval of the relative regression model. * = *p* < 0.05.

Lastly, an exploratory analysis was conducted to examine whether baseline HR predicted the viewing time or zHR trends within each cluster. Linear regression models were employed between Mann–Kendall *τ* values and baseline HR. Interestingly, baseline HR significantly predicted the zHR trend in Cluster 1 (β = −0.0091, 95% CI [−0.0169, −0.0013], R^2^ = 0.472, *p* = 0.028) and the viewing time trend in Cluster 3 (β = −0.0173, 95% CI [−0.0339, −0.0007], R^2^ = 0.222, *p* = 0.042) (see Figure 7).

## 4. Discussion

In this study, we aimed (1) to determine whether museum fatigue could emerge during a visit to the ETRU museum in Rome, (2) to assess whether heart rate could represent a reliable implicit measure of museum fatigue, and (3) to test whether the emergence of museum fatigue may be modulated by visitors’ personal characteristics. To fulfill these goals, we investigated changes in viewing time and heart rate with visit progression and their associations with the visitors’ perceived emotional state after the visit. Moreover, we explored whether these changes and associations differed when taking into account visitors’ personal characteristics. In line with the predictions, we observed a decreasing trend in viewing time and an increasing trend in heart rate values during visit progression. These findings suggest that museum fatigue occurred during the ETRU visit and that heart rate values may represent a novel implicit measure of the phenomenon. In addition, different correlations between visit time, mean heart rate, and emotional state ratings emerged within each cluster. While these results should be interpreted cautiously due to the relatively small sample size of the three clusters, they suggest that the underlying mechanisms of museum fatigue may be modulated by visitors’ personal characteristics. The following paragraphs discuss these results in greater depth.

### 4.1. The Presence of Museum Fatigue During the Visit at ETRU

When considering all 45 participants, viewing time decreased progressively over the course of the visit, suggesting the emergence of fatigue during the ETRU experience. This result appears robust, as it emerged in the majority of participants when individual analyses were conducted. Moreover, visit time was negatively associated with involvement, calmness, and enthusiasm ratings at the end of the visit. This is in line with previous studies that linked short visit time and negative emotional state with museum fatigue [1]. These results were expected given the environmental characteristics of the ETRU visit. First, the visit lasted on average 80 min, substantially exceeding the typical duration of visitor attention to exhibitions, which is less than 20 min [21]. According to the attention–value model proposed by [22], visitors are motivated to pay attention to an artifact when its value is high. An artifact’s value is high when the ratio between benefits (utility/satisfaction) and costs (time/effort) is positive. In the initial stages of the visit, visitors spend more time in each area as they are implicitly attracted by a wide range of stimuli, a process called capture of automatic attention [22,23]. When fatigue occurs, visitors focus on fewer artifacts by using goal-directed voluntary attention [22,23]. This process increases the cognitive resource demand of each artifact, reducing their value and thus the viewing time [24,25]. Moreover, many areas featured a very large number of homologous artifacts. This repetition is known to generate a phenomenon associated with museum fatigue called satiation or hedonic decline, that is, the habituation response induced by repeated exposure to similar stimuli, reflected by decreased interest and viewing time [11,12,26,27]. Lastly, the visit at ETRU lacked rest areas, which, according to optimum stimulation level theory, can increase the likelihood of satiation [11,28,29].

### 4.2. Heart Rate as an Implicit Measure of Museum Fatigue

Across all 45 participants, heart rate values increased progressively over the course of the visit. Again, the result was robust, as it emerged consistently at individual level. Moreover, the average heart rate during the visit was negatively associated with both visit time and emotional state ratings. Therefore, the increase in heart rate may represent an increase in arousal (sympathetic activation) induced by decreased emotional state, rather than a positive emotional response related to the visit. This is in line with previous studies employing psychophysiological measures to assess the attention of participants and their interest in specific artifacts [13,14,15,16]. Indeed, sympathetic activation has been associated with low perception of aesthetic quality [16], and a longer time spent in a museum has been related to decreased emotional arousal [13]. Taken together, these results suggest that decreasing viewing time and increasing heart rate as a museum visit progresses may reflect museum fatigue, particularly in relation to diminished motivation and interest in continuing the visit. This indicates that not only viewing time, but also implicit measures of arousal, such as heart rate, may represent valuable measures of the phenomenon during museum visits. However, it must be noted that, especially in ecological contexts, heart rate fluctuations may also reflect physical movement or cognitive load, which may not be related to museum fatigue. Furthermore, both branches of the autonomic nervous system (ANS) contribute to heart rate, restricting the possible interpretations on these results. To better understand the observed physiological changes, future investigations should employ physiological measures that are specifically linked to the sympathetic or the parasympathetic activity. For instance, electrodermal activity (EDA) is known to be exclusively modulated by the sympathetic branch of ANS [18,30]. Conversely, heart rate variability (HRV) indices, such as root mean squared values of successive differences or high-frequency power bands, are known to be specifically linked with parasympathetic activity [31]. Another interesting approach would be to investigate the change in balance between the two branches by employing the normalized high-frequency index of HRV [31,32]. To do so, future studies should employ devices that are more sensitive and with higher sample rates.

### 4.3. The Role of Personal Factors in Modulating the Emergence of Museum Fatigue

When examining specific participant clusters, the decreasing viewing time trend was found in all three visitor groups. This suggests that the environmental factors present during the visit—long visit, great number of artifacts, no rest areas—may have played a primary role in the emergence of the museum fatigue, independently from visitors’ personal characteristics. Conversely, differences between clusters were found in heart rate values and in the association between emotional state and both visit time and heart rate. Moreover, baseline heart rate values were found to shape these trends in some clusters. These results suggest that environmental characteristics play a role in the emergence of museum fatigue across all participants, but that the underlying mechanisms of museum fatigue may vary across visitor clusters, potentially due to differences in their individual characteristics.

In particular, adults classified as the general public (Cluster 1) showed a positive correlation between relaxation ratings and visit time, and an increasing heart rate trend during the visit. Also, the visit time trend was more pronounced in those visitors who demonstrated low heart rate values at rest. These results indicate that museum fatigue emerged in these visitors even though the positive correlation between visit time and relaxation suggests that they appreciated the visit. Thus, environmental factors and baseline physiological values may have contributed to the emergence of museum fatigue in this cluster, and positive emotional states did not mitigate these effects. The case of highly educated participants classified as museumgoers (Cluster 2) is different. Indeed, in these visitors, an increasing heart rate trend was observed, and their heart rate during the visit was strongly negatively correlated with visit time and with perceived involvement, calmness, and enthusiasm. Hence, we may hypothesize that the environmental characteristics of ETRU did not match the expectations of these experienced visitors, leading to negative physiological and emotional responses that consequently reduced the visit time. Therefore, in this visitor cluster, both environmental and personal factors may have contributed to the emergence of museum fatigue. Lastly, visitors classified as non-public (Cluster 3) showed no heart rate trends but showed a negative association between visit time and perceived relaxation. These results may be expected by visitors who were less motivated to visit the museum, as they demonstrated increased relaxation, but only in the case of short visits. This is consistent with the steeper decline in viewing time observed in those visitors in Cluster 3 who demonstrated high sympathetic activity at rest. Indeed, this parameter has been linked with low perception of aesthetic quality [16], but also with difficulties in maintaining attention [33]. Therefore, in those visitors, the reduction in artifact value and the use of voluntary attention described by the attention–value model [23] may have appeared rapidly, decreasing viewing time without generating negative emotions. Thus, the characteristics of the ETRU visit may have also exacerbated fatigue in these visitors due to their initial lack of interest in the visit, again suggesting that both environmental and personal factors contributed to museum fatigue. Hence, future studies should take into account visitors’ individual characteristics when drawing conclusions from viewing time results, as they may influence the psychophysiological responses to the environmental characteristics of the museum visit. However, the results emerging from single clusters should be interpreted cautiously due to some limitations. First, the cluster sample sizes are small (fewer than 20 participants), potentially limiting the reliability of the observed results. Moreover, while sex, age, education, and type of public represent interesting variables for participant clustering, other variables are similarly important and yet were not considered in the present study. For instance, future studies should investigate whether visiting alone or in groups may modulate the emergence of museum fatigue, or whether prior visits may facilitate or diminish the occurrence of this phenomenon.

### 4.4. Practical Implications

Some practical implications for museum exhibition design may originate from the findings of the present study. First, environmental characteristics should be adjusted to preserve the perceived value of individual artifacts throughout the visit. To achieve this, the number of displayed artifacts should be reduced and aligned to the physiological decline of automatic attention. For long visits, rest areas should also be included and strategically placed. This approach would sustain visitors’ engagement while counteracting satiation effects and reducing the emergence of museum fatigue. In addition, the results of this study, although limited by the relatively small sample size, suggest the importance of tailoring the visitor experience to personal characteristics. Pre-visit questionnaires could be used to assess visitors’ interest and expertise, allowing the provision of customized content, visit duration, routes, or interaction modes. This personalized approach could better align exhibition features with visitor expectations, thereby optimizing engagement, and reducing the onset of fatigue across different visitor profiles. However, these implications are derived from results restricted to the ETRU exhibition and are not fully generalizable due to the lack of a control condition. Future investigations should be conducted in different settings and with larger sample sizes to confirm these findings. Moreover, although the tracking system used in this study determined the visitors’ entry and exit time in each museum area, it could not specify their exact location within each area. Future investigations should employ more precise tracking systems to triangulate visitors’ position, enabling the visualization of visiting trajectories, stopping duration, and physiological changes in specific locations. This would provide deeper insights into the visitors’ behavior throughout the visit, further refining the proposed systems aimed at tailoring the experience to individual visitors.

## 5. Conclusions

Despite some limitations, the study conducted at ETRU confirmed that environmental characteristics—long visits, great number of artifacts and absence of rest areas—induce museum fatigue in visitors. Moreover, it revealed that heart rate may be employed as an objective and implicit measure of museum fatigue. Lastly, the findings of this study revealed that the emergence of museum fatigue may be modulated by visitors’ personal characteristics, emphasizing the relevance of these factors for future research on museum fatigue and for the planning of museum exhibitions.

## Figures and Tables

**Table 1:** Demographic data of participants.

Demographic Variable	*n*	%
**Sex**		
Female	29	35.56
Male	16	64.44
**Age**		
Young (18–35)	17	37.78
Adult (36–64)	15	33.33
Old (65+)	13	28.89
**Education**		
Middle/High school	19	42.22
Bachelor’s/Master’s Degree	26	57.78
**Public**		
Non-public	10	22.22
Public	16	35.56
Museumgoers	19	42.22

**Table 2:** Dimensions of each museum area.

Area	Surface (m^2^)
A1	118.8
A2	301
A3	49
A4	36.8
A5	515.5
A6	515.9
A7	58.1
A8	271.8
A9	554.36
**Total**	**2421.24**

**Table 3:** Demographic data of participants per cluster.

Demographic Variable	Cluster 1		Cluster 2		Cluster 3	
	*n*	%	*n*	%	*n*	%
**Sex**						
Female	15	78.95	8	50.00	6	60.00
Male	4	21.05	8	50.00	4	40.00
**Age**						
Young (18–35)	5	26.32	10	62.50	2	20.00
Adult (36–64)	12	63.16	6	37.50	3	30.00
Old (65+)	3	10.52	2	20.00	4	50.00
**Education**						
Middle/High school	11	57.89	2	12.50	6	60.00
Bachelor’s/Master’s Degree	8	42.11	14	87.50	4	40.00
**Public**						
Non-public	0	0.00	0	0.00	10	100.00
Public	15	78.95	1	6.25	0	0.00
Museumgoers	4	21.05	15	93.75	0	0.00

## Data Availability

The original data presented in the study are openly available in Mendeley Data at doi: 10.17632/x8v2wt22gr.1.

## Abbreviations

The following abbreviations are used in this manuscript:

AR	Augmented Reality
BLE	Bluetooth Low Energy
BVP	Blood Volume Pulse
HCA	Hierarchical Cluster Analysis
HR	Heart Rate
MCA	Multiple Correspondence Analysis

## Appendix A

**Table A1:** Individual trends in viewing time and zHR and individual correlations between viewing time and zHR.

Participant	Viewing Time Trend (*τ*)	zHR Trend (*τ*)	Viewing Time-zHR Correlation (ρ)	Viewing Time-zHR Correlation (*p*)
**Cluster 1**				
1	−0.78	0.17	−0.08	0.83
4	−0.56	0.33	−0.45	0.22
6	−0.50	−0.44	0.28	0.46
11	−0.89	0.28	−0.15	0.70
13	−0.67	0.28	−0.47	0.21
16	−0.83	0.28	−0.57	0.11
17	−0.44	−0.44	0.23	0.55
18	−0.56	0.33	−0.58	0.10
20	−0.11	−0.28	−0.55	0.12
23	−0.89	0.56	−0.85	0.00
24	−0.89	0.28	−0.35	0.36
26	−0.17	0.22	0.23	0.55
27	−0.61	0.39	−0.82	0.01
33	−0.33	−0.11	−0.03	0.93
34	−0.67	0.22	−0.48	0.19
36	−0.33	0.28	−0.27	0.49
45	−0.28	0.11	−0.10	0.80
57	−0.39	−0.33	−0.15	0.70
58	−0.67	0.67	−0.80	0.01
**Cluster 2**				
8	−0.67	0.33	−0.18	0.64
9	−0.33	−0.17	0.40	0.29
10	−0.61	−0.22	0.10	0.80
41	−0.50	0.28	−0.67	0.05
42	0.28	−0.44	−0.17	0.67
43	−0.44	0.22	−0.68	0.04
44	−0.61	0.44	−0.33	0.38
48	−0.94	0.44	−0.70	0.04
49	−0.72	0.39	−0.23	0.55
50	−0.22	0.44	0.15	0.70
53	−0.50	0.67	−0.47	0.21
54	−0.61	0.67	−0.52	0.15
55	−0.67	0.22	−0.62	0.08
56	−0.44	0.28	−0.85	0.00
60	−0.72	−0.33	0.17	0.67
61	−0.56	0.06	0.00	1.00
**Cluster 3**				
15	−0.44	−0.50	0.03	0.93
19	−0.44	0.00	−0.37	0.33
25	−0.56	−0.22	0.48	0.19
31	−0.17	0.22	−0.37	0.33
32	−0.33	0.00	0.02	0.97
35	−0.72	0.44	−0.87	0.00
37	−0.44	−0.17	0.35	0.36
38	−0.44	0.11	−0.30	0.43
52	−0.67	0.17	−0.12	0.77
